# Supplementary material for: GCAF(TMEM251) regulates lysosome biogenesis by activating the mannose-6-phosphate pathway
Source: Nat Commun. 2022 Sep 12;13:5351. doi: 10.1038/s41467-022-33025-1 (PMC9468337; doi:10.1038/s41467-022-33025-1)
Supplement: Supplementary file 1 — Supplementary Information [file 41467_2022_33025_MOESM1_ESM.pdf]

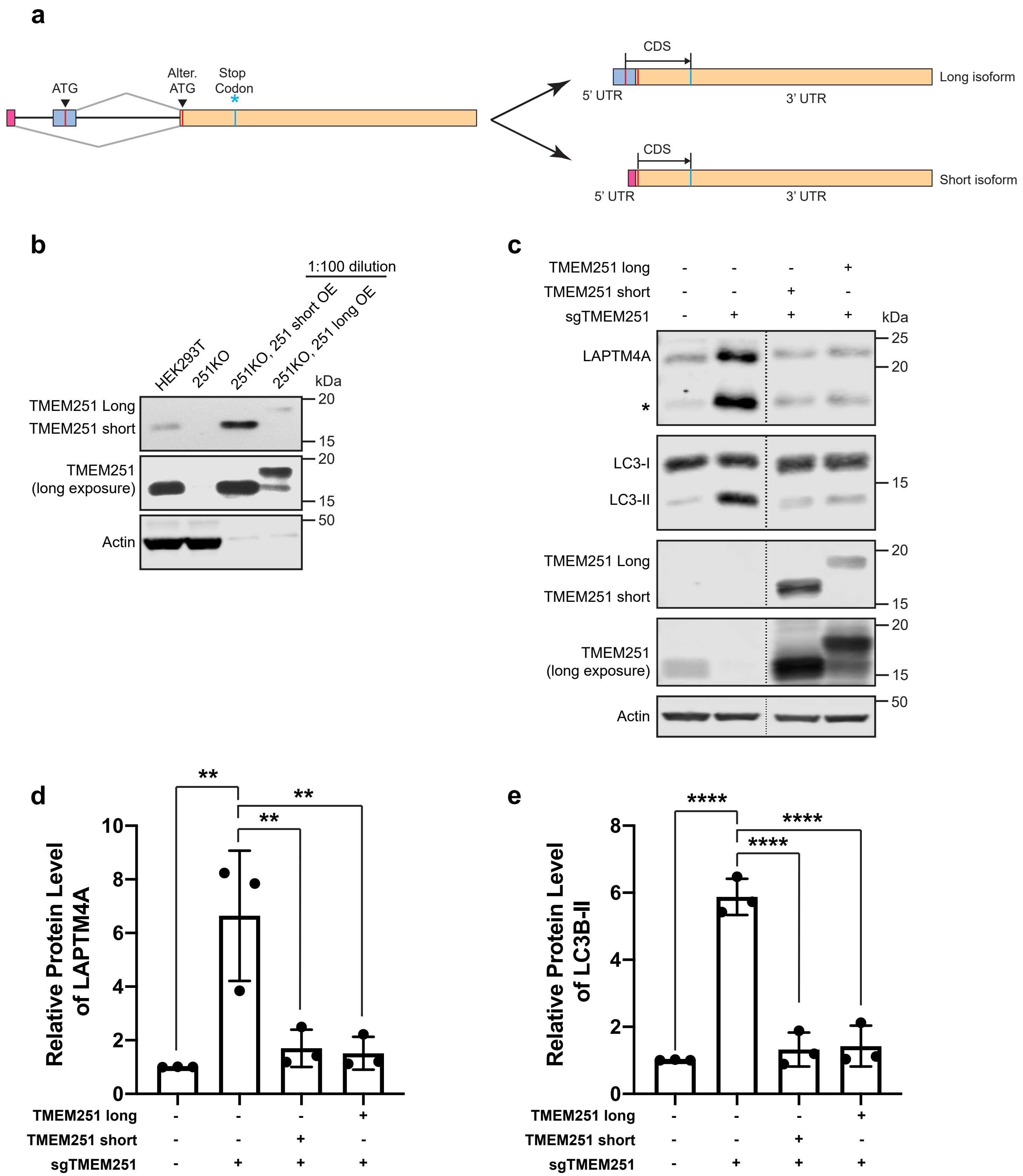
a
ATG
Alter. ATG
Stop Codon
CDS
5' UTR
3' UTR
Long isoform
Short isoform
b
1:100 dilution
HEK293T
251KO
251KO, 251 short OE
251KO, 251 long OE
kDa
TMEM251 Long
TMEM251 short
TMEM251 (long exposure)
Actin
20
15
50
c
TMEM251 long
TMEM251 short
sgTMEM251
LAPTM4A
*
LC3-I
LC3-II
TMEM251 Long
TMEM251 short
TMEM251 (long exposure)
Actin
25
20
15
50
d
Relative Protein Level of LAPTM4A
**
10
8
6
4
2
0
TMEM251 long
TMEM251 short
sgTMEM251
e
Relative Protein Level of LC3B-II
****
8
6
4
2
0
TMEM251 long
TMEM251 short
sgTMEM251

Supplementary Figure 1: Transcriptional variants of TMEM251. (a) A schematic representation of the alternative splicing of TMEM251 mRNA. **(b)** Endogenous TMEM251 and the overexpression of different isoforms of TMEM251 in HEK293T cells (n=3 independent replicates). The overexpression samples were diluted to make them comparable to the endogenous TMEM251. **(c)** Overexpression of either short or long isoform of TMEM251 rescues LAPTM4A and LC3B-II protein levels in TMEM251-deficient cells. * highlights the cleavage product of LAPTM4A. **(d)** Quantification of LAPTM4A protein level in c. Mean of n=3 independent replicates is shown. Error bars represent standard deviation. **: $p \leq 0.01$. **(e)** Quantification of LC3B-II protein level in c. Mean of n=3 independent replicates is shown. Error bars represent standard deviation. ****: $p \leq 0.0001$. See source data file for exact P values. Related to Figure 2.

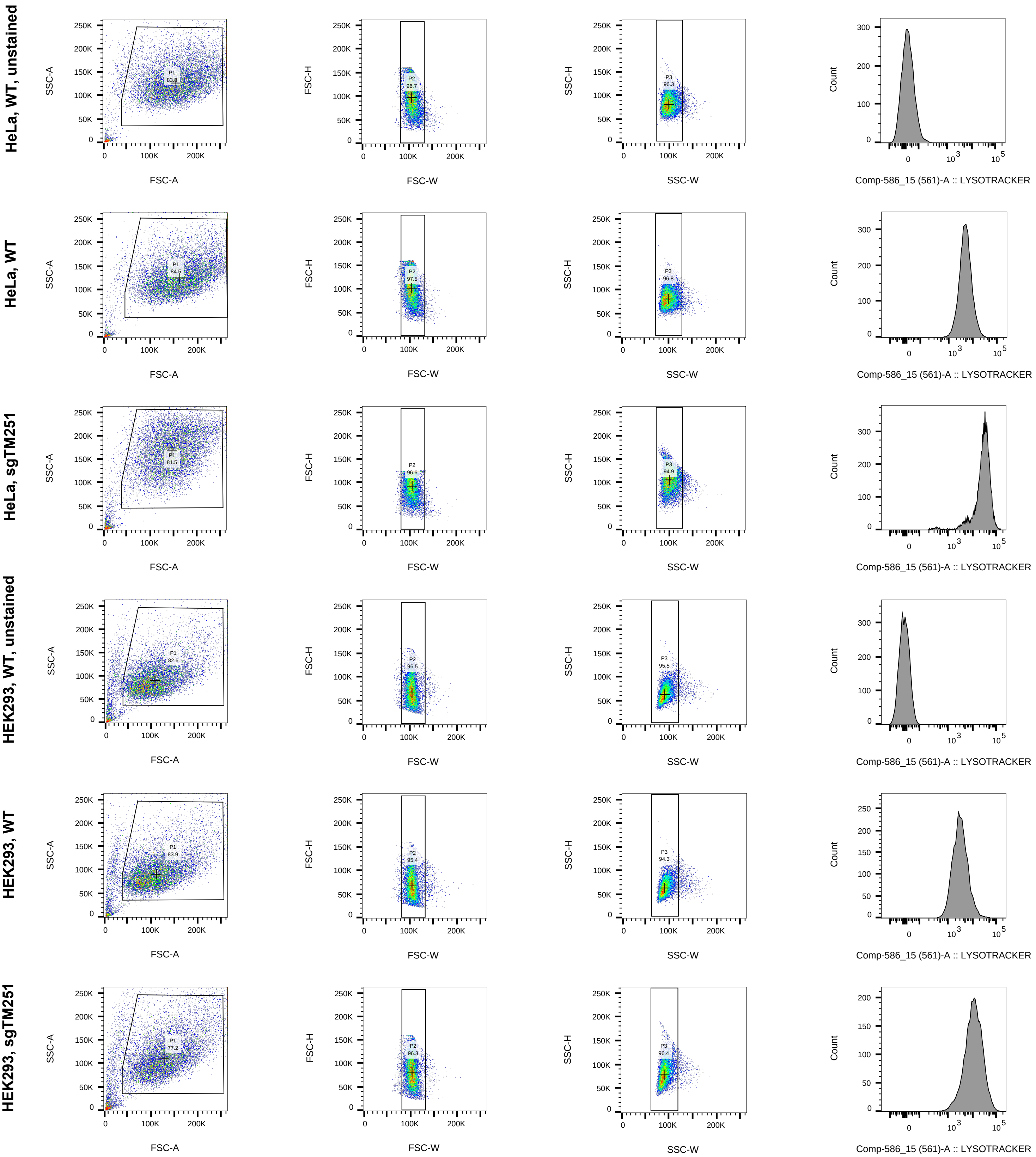
HeLa, WT, unstained
HeLa, WT
HeLa, sgTM251
HEK293, WT, unstained
HEK293, WT
HEK293, sgTM251
SSC-A
FSC-A
FSC-H
FSC-W
SSC-H
SSC-W
Count
Comp-586_15 (561)-A :: LYSOTRACKER

Supplementary Figure 2: Gating strategy for the flow cytometry analysis after LysoTracker staining. Related to Figure 3.

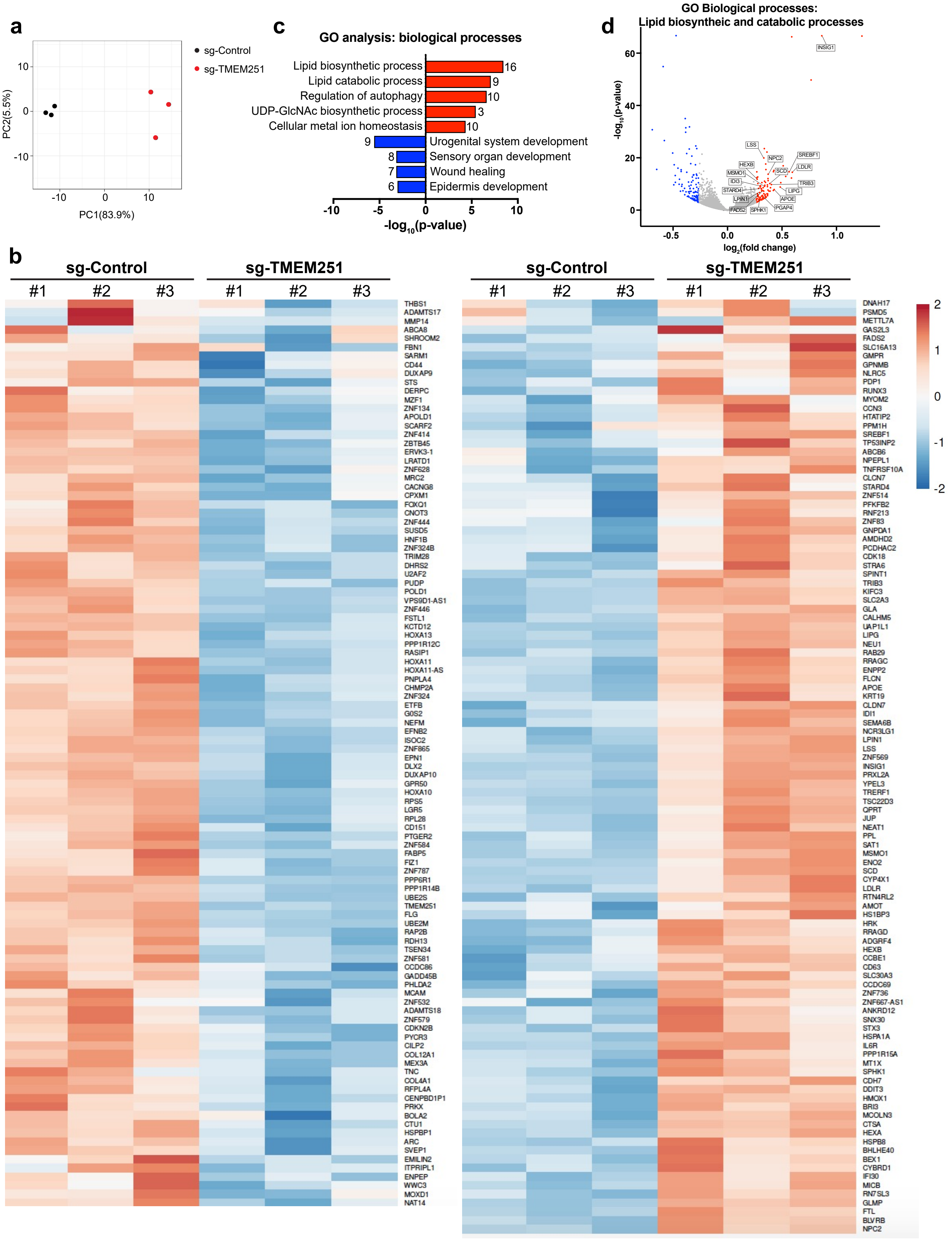
a
sg-Control
sg-TMEM251
PC2(5.5%)
PC1(83.9%)
10
0
-10
-10
0
10
c
GO analysis: biological processes
Lipid biosynthetic process 16
Lipid catabolic process 9
Regulation of autophagy 10
UDP-GlcNAc biosynthetic process 3
Cellular metal ion homeostasis 10
9 Urogenital system development
8 Sensory organ development
7 Wound healing
6 Epidermis development
-10
-5
0
5
10
$-\log_{10}$(p-value)
d
GO Biological processes:
Lipid biosynthetic and catabolic processes
$-\log_{10}$(p-value)
$\log_2$(fold change)
60
40
20
0
-0.5
0.0
0.5
1.0
INSIG1
LSS
NPC2
SREBF1
HEXB
MSMO1
SCD
LDLR
IDI3
TRIB3
STARD4
LIPG
LPIN1
APOE
FADS2
SPHK1
PGAP4
b
sg-Control
sg-TMEM251
#1
#2
#3
#1
#2
#3
THBS1
ADAMTS17
MMP14
ABCA8
SHROOM2
FBN1
SARM1
CD44
DUXAP9
STS
DERPC
MZF1
ZNF134
APOLD1
SCARF2
ZNF414
ZBTB45
ERVK3-1
LRATD1
ZNF628
MRC2
CACNG8
CPXM1
FOXQ1
CNOT3
ZNF444
SUSD5
HNF1B
ZNF324B
TRIM28
DHRS2
U2AF2
PUDP
POLD1
VPS9D1-AS1
ZNF446
FSTL1
KCTD12
HOXA13
PPP1R12C
RASIP1
HOXA11
HOXA11-AS
PNPLA4
CHMP2A
ZNF324
ETFB
G0S2
NEFM
EFNB2
ISOC2
ZNF865
EPN1
DLX2
DUXAP10
GPR50
HOXA10
RPS5
LGR5
RPL28
CD151
PTGER2
ZNF584
FABP5
FIZ1
ZNF787
PPP6R1
PPP1R14B
UBE2S
TMEM251
FLG
UBE2M
RAP2B
RDH13
TSEN34
ZNF581
CCDC86
GADD45B
PHLDA2
MCAM
ZNF532
ADAMTS18
ZNF579
CDKN2B
PYCR3
CILP2
COL12A1
MEX3A
TNC
COL4A1
RFPL4A
CENPBD1P1
PRKX
BOLA2
CTU1
HSPBP1
ARC
SVEP1
EMILIN2
ITPRIPL1
ENPEP
WWC3
MOXD1
NAT14
sg-Control
sg-TMEM251
#1
#2
#3
#1
#2
#3
DNAH17
PSMD5
METTL7A
GAS2L3
FADS2
SLC16A13
GMPR
GPNMB
NLRC5
PDP1
RUNX3
MYOM2
CCN3
HTATIP2
PPM1H
SREBF1
TP53INP2
ABCB6
NPEPL1
TNFRSF10A
CLCN7
STARD4
ZNF514
PFKFB2
RNF213
ZNF83
GNPDA1
AMDHD2
PCDHAC2
CDK18
STRA6
SPINT1
TRIB3
KIFC3
SLC2A3
GLA
CALHM5
UAP1L1
LIPG
NEU1
RAB29
RRAGC
ENPP2
FLCN
APOE
KRT19
CLDN7
IDI1
SEMA6B
NCR3LG1
LPIN1
LSS
ZNF569
INSIG1
PRXL2A
YPEL3
TRERF1
TSC22D3
QPRT
JUP
NEAT1
PPL
SAT1
MSMO1
ENO2
SCD
CYP4X1
LDLR
RTN4RL2
AMOT
HS1BP3
HRK
RRAGD
ADGRF4
HEXB
CCBE1
CD63
SLC30A3
CCDC69
ZNF736
ZNF667-AS1
ANKRD12
SNX30
STX3
HSPA1A
IL6R
PPP1R15A
MT1X
SPHK1
CDH7
DDIT3
HMOX1
BRI3
MCOLN3
CTSA
HEXA
HSPB8
BHLHE40
BEX1
CYBRD1
IFI30
MICB
RN7SL3
GLMP
FTL
BLVRB
NPC2
2
1
0
-1
-2

Supplementary Figure 3: RNA sequencing analysis of sgTMEM251 cells vs. control cells. **(a)** Principal component analysis (PCA) of RNA-sequencing. **(b)** A heatmap of 211 DEGs altered in sgTMEM251 vs. control cells. **(c)** GO biological processes analysis of DEGs altered in sgTMEM251 (n=3 independent replicates) vs. control (n=3 independent replicates) cells. The number of genes in each pathway is indicated. **(d)** A Volcano plot of RNA-seq analysis of sgTMEM251 (n=3 independent replicates) vs. control (n=3 independent replicates) cells. Annotated genes are classified in lipid biosynthetic and catabolic processes. Related to Figure 3.

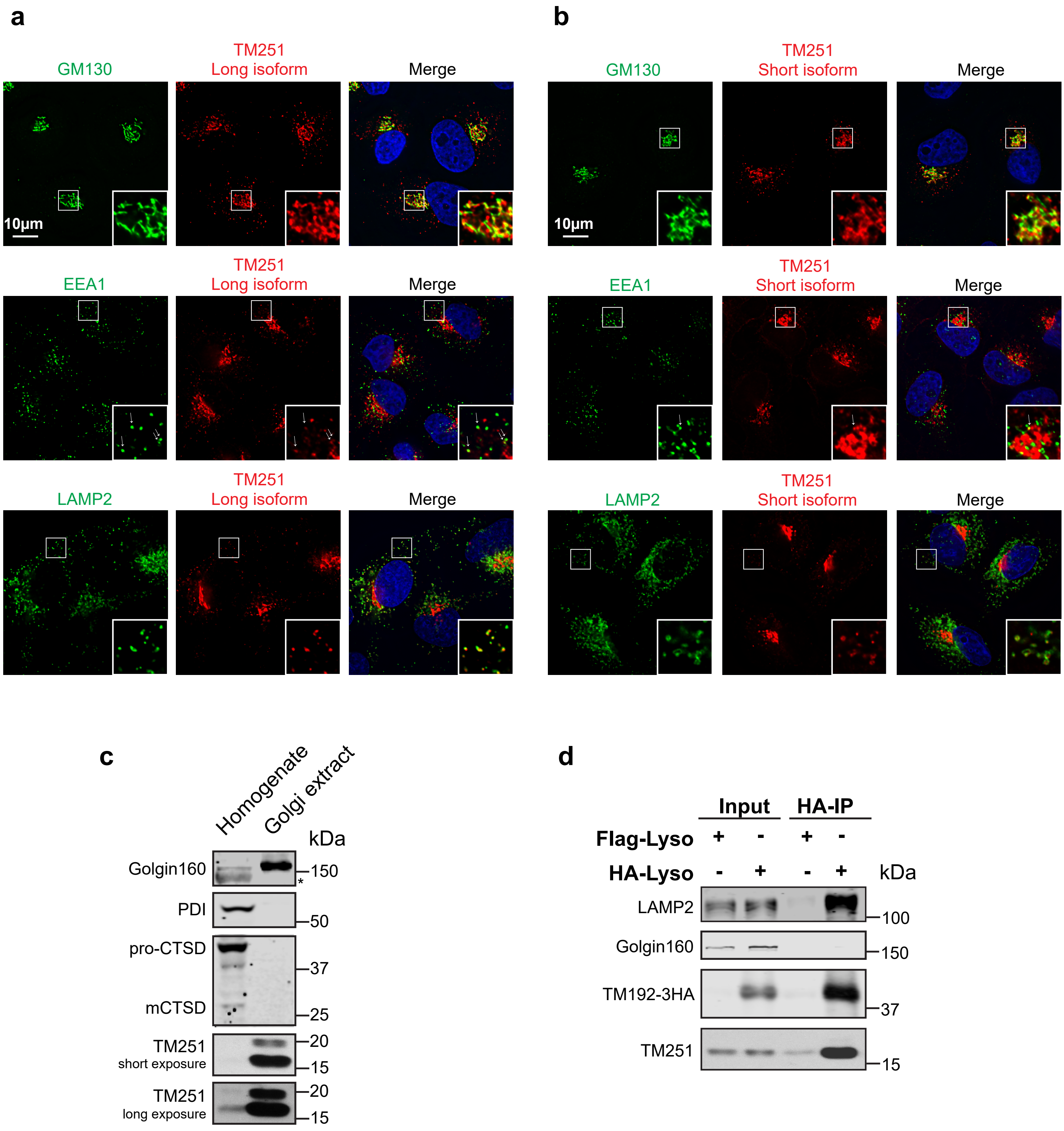
a
GM130
TM251
Long isoform
Merge
10μm
EEA1
TM251
Long isoform
Merge
LAMP2
TM251
Long isoform
Merge
b
GM130
TM251
Short isoform
Merge
10μm
EEA1
TM251
Short isoform
Merge
LAMP2
TM251
Short isoform
Merge
c
Homogenate
Golgi extract
kDa
Golgin160
150
*
PDI
50
pro-CTSD
37
mCTSD
25
TM251
short exposure
20
15
TM251
long exposure
20
15
d
Input
HA-IP
Flag-Lyso + - + -
HA-Lyso - + - +
kDa
LAMP2
100
Golgin160
150
TM192-3HA
37
TM251
15

Supplementary Figure 4: Subcellular localization of TMEM251. (a-b) Immunofluorescence showing the localization of overexpressed long and short isoform of TMEM251 in HeLa cells (n=50 cells from three independent replicates) co-stained with GM130, EEA1, and LAMP2. Scale bar: 10 µm. **(c)** Endogenous TMEM251 in rat liver Golgi extract. n=2 independent replicates. **(d)** Endogenous TMEM251 in purified lysosomes from HEK293T cells. n=3 independent replicates. Related to Figure 5.

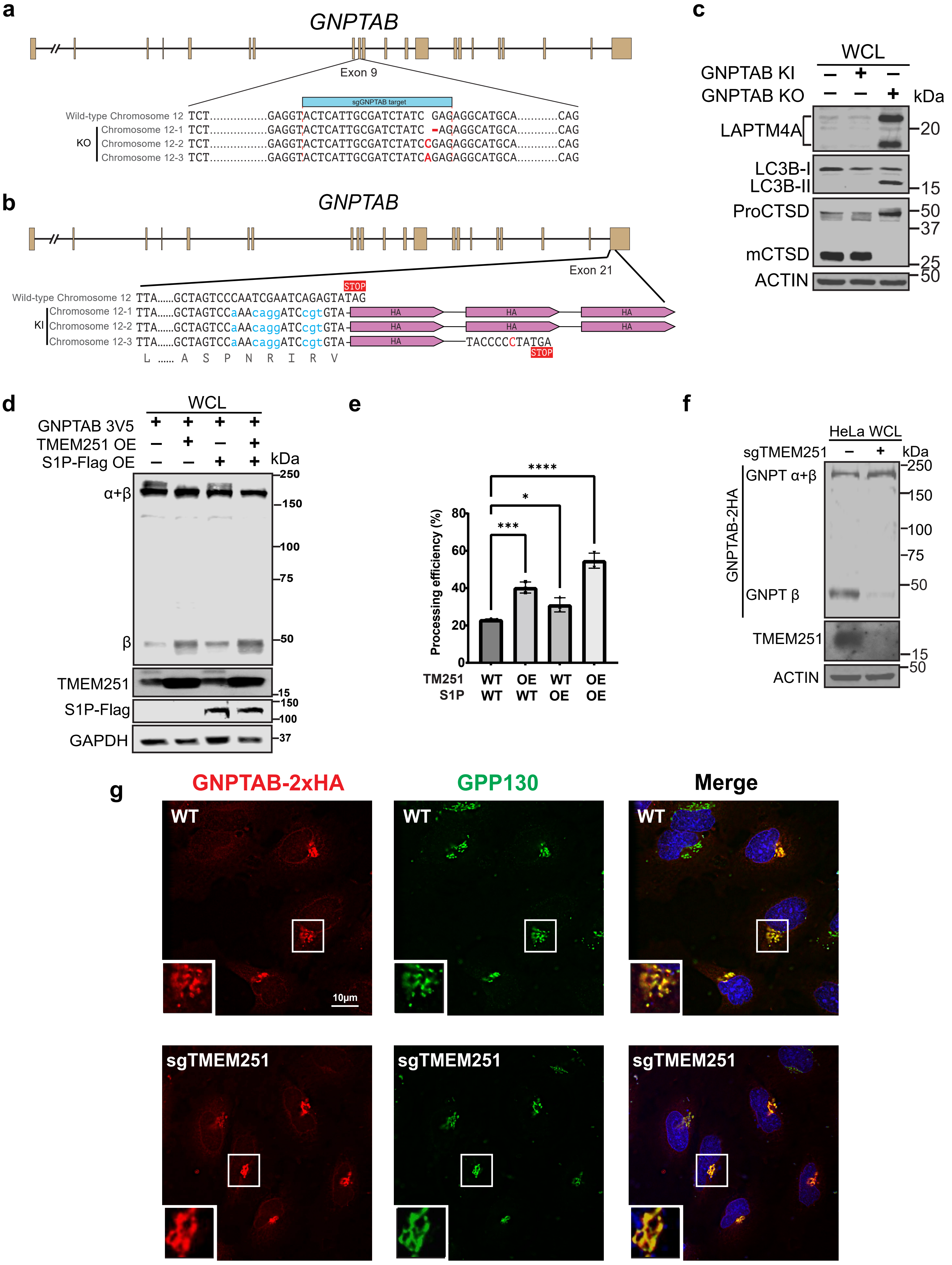

a
GNPTAB
Exon 9
sgGNPTAB target
Wild-type Chromosome 12 TCT................GAGGTACTCATTGCGATCTATC GAGAGGCATGCA...........CAG
KO
Chromosome 12-1 TCT................GAGGTACTCATTGCGATCTATC -AGAGGCATGCA...........CAG
Chromosome 12-2 TCT................GAGGTACTCATTGCGATCTATCCGAGAGGCATGCA...........CAG
Chromosome 12-3 TCT................GAGGTACTCATTGCGATCTATCAGAGAGGCATGCA...........CAG
b
GNPTAB
Exon 21
STOP
Wild-type Chromosome 12 TTA......GCTAGTCCCAATCGAATCAGAGTATAG
KI
Chromosome 12-1 TTA......GCTAGTCCaAAcaggATCcgtGTA
Chromosome 12-2 TTA......GCTAGTCCaAAcaggATCcgtGTA
Chromosome 12-3 TTA......GCTAGTCCaAAcaggATCcgtGTA
HA
TACCCCCTATGA
STOP
L ...... A S P N R I R V
c
WCL
GNPTAB KI
GNPTAB KO
kDa
LAPTM4A
LC3B-I
LC3B-II
ProCTSD
mCTSD
ACTIN
20
15
50
37
25
50
d
WCL
GNPTAB 3V5
TMEM251 OE
S1P-Flag OE
kDa
α+β
β
TMEM251
S1P-Flag
GAPDH
250
150
100
75
50
15
150
100
37
e
****
*
***
Processing efficiency (%)
80
60
40
20
0
TM251 WT OE WT OE
S1P WT WT OE OE
f
HeLa WCL
sgTMEM251
kDa
GNPTAB-2HA
GNPT α+β
GNPT β
TMEM251
ACTIN
250
150
100
75
50
15
50
g
GNPTAB-2xHA
GPP130
Merge
WT
10μm
sgTMEM251

Supplementary Figure 5: TMEM251 functions at the cleavage step of GNPT α/β precursor by S1P. (a) Sequencing analysis to confirm the genotype of GNPTAB single colony KO HEK293T cells. All three chromosomes from the same single colony cell contain either 1 bp deletion or insertion (highlighted in red), which leads to a frameshift, indicating a complete KO of GNPTAB protein. **(b)** Sequencing analysis of the GNPTAB-3xHA endogenous knock-in single colony cells. Two chromosomes from the same single colony cell contain 3x HA insertion, while the third chromosome only contains 1x HA insertion due to a premature stop codon generated during recombination. Sequences highlighted in blue lower case showed the silencing mutations designed in the KI template to prevent the cutting from Cas9. Sequence highlighted in red showed one bp insertion, which leads to a premature stop codon in the second HA region. **(c)** Analysis of lysosome function in GNPTAB KO and GNPTAB-3xHA KI cells (n=3 independent replicates). **(d)** Overexpression of TMEM251 stimulated the processing of GNPTAB-3V5. **(e)** Quantification of GNPTAB-3V5 processing efficiency in d. Mean of n=3 independent replicates is shown. Error bars represent standard deviation. *: $p \leq 0.05$, *** $p \leq 0.001$, ****: $p \leq 0.0001$. See source data file for exact P values. **(f)** The processing of overexpressed GNPTAB-2xHA in WT and TMEM251 KO HeLa cells (n=3 independent replicates). **(g)** Immunofluorescence showing the localization of overexpressed GNPTAB-2xHA in WT and TMEM251 KO HeLa cells (n=50 cells from three independent replicates) co-stained with GPP130. Scale bar: 10 µm. Related to Figure 6.

Supplementary Table 1: Hypersecreted lysosomal enzymes in TMEM251-deficient cells. $Log_2FC>1$, $p<0.05$.

AGA	ARSA	ARSK	C3	CPQ
CREG1	CTSA	CTSB	CTSC	CTSH
CTSL	CTSV	DNASE2	EPDR1	GAA
GBA	GGH	GLB1	GNS	GRN
GUSB	HEXA	HEXB	IDS	LGMN
LIPA	MAN2B1	MAN2B2	MANBA	NAGLU
NEU1	NPC2	PLBD2	PRCP	PSAP
RNASET2	SGSH	SIAE	TPP1	

**Supplementary Table 2: Mammalian cell lines used in this study**		
Cell lines	***Description***	***reference/source***
Human HEK293	CRL-1573	ATCC
Human HEK293T	CRL-3216	ATCC
Human HeLa	CCL-2	ATCC
Human HEK293, GFP-RNF152	pHAGE2-EF1α-EGFP-RNF152-IRES-Puro	Zhang et al., 2021
Human HEK293, GFP-RNF152	pHAGE2-EF1α-EGFP-RNF152-IRES-mCherry	Zhang et al., 2021
Human HEK293, GFP-RNF152, Cas9	pHAGE2-EF1α-EGFP-RNF152-IRES-mCherry, LentiCas9-Blast	This study
Human HEK293, sg1-TMEM251	Polyclonal TMEM251 CRISPR-Cas9 KO with sgRNA-1	This study
Human HEK293, sg2-TMEM251	Polyclonal TMEM251 CRISPR-Cas9 KO with sgRNA-1	This study
Human HEK293, sg3-TMEM251 (for RNA-seq)	Polyclonal TMEM251 CRISPR-Cas9 KO with sgRNA-3	This study
Human HEK293, sg1-TMEM251, TMEM251 long isoform	Polyclonal TMEM251 CRISPR-Cas9 KO with sgRNA-1, pLenti6.3-TMEM251(long)-BLAST	This study
Human HEK293 sg1-TMEM251, TMEM251 short isoform	Polyclonal TMEM251 CRISPR-Cas9 KO with sgRNA-1, pLenti6.3-TMEM251(short)-BLAST	This study
Human HEK293T, TMEM251KO	CRISPR-Cas9 KO of TMEM251, sgRNA-1, single colony.	This study
Human HEK293T, sg-TMEM251	Polyclonal TMEM251 CRISPR-Cas9 KO with sgRNA-1	This study
Human HEK293T, TMEM251KO, TMEM251 long isoform	TMEM251 CRISPR-Cas9 KO, pLenti6.3-TMEM251(long)-BLAST	This study
Human HEK293T, TMEM251KO, TMEM251 short isoform	TMEM251 CRISPR-Cas9 KO, pLenti6.3-TMEM251(short)-BLAST	This study
Human HEK293T, FLAG-Lyso	pLJC5-TMEM192-2XFLAG-Puro (Addgene 102929)	This study (Abu-Remaileh et al. 2017)

Human HEK293T, HA-Lyso	pLJC5-TMEM192-3XHA-Puro (Addgene 102930)	This study (Abu-Remaileh et al. 2017)
Human HEK293T, GNPTAB KO	CRISPR-Cas9 KO of GNPTAB, single colony.	This study
Human HEK293T, CI-MPR KO	CRISPR-Cas9 KO of CI-MPR, single colony.	This study
Human HEK293T, CI-MPR KO, TMEM251KO	CRISPR-Cas9 KO of CI-MPR and TMEM251, single colony.	This study
Human HEK293T, GNPTAB-3HA knockin	CRISPR-Cas9 knockin of 3HA tag the C-terminus of GNPTAB	This study
Human HEK293T, GNPTAB-3HA knockin, TMEM251KO	CRISPR-Cas9 knockin of 3HA tag the C-terminus of GNPTAB, CRISPR-Cas9 KO of TMEM251	This study
Human HEK293T, GNPTAB-3HA knockin, TMEM251KO, TMEM251OE	CRISPR-Cas9 knockin of 3HA tag the C-terminus of GNPTAB, CRISPR-Cas9 KO of TMEM251, pLenti6.3-TMEM251(short)	This study
Human HeLa, TMEM251KO #1	CRISPR-Cas9 KO of TMEM251, sgRNA-1, single colony #1	This study
Human HeLa, TMEM251KO #2	CRISPR-Cas9 KO of TMEM251, sgRNA-2, single colony #2	This study
Human HeLa, sgTMEM251	Polyclonal TMEM251 CRISPR-Cas9 KO with sgRNA-1	This study
Human HeLa, TMEM251 long isoform	pLenti6.3-TMEM251(long)-BLAST	This study
Human HeLa, TMEM251 short isoform	pLenti6.3-TMEM251(short)-BLAST	This study

**Supplementary Table 3: Mammalian plasmids used in this study**			
Vector	***Insert***	***description***	***reference/source***
pHEK293Ultra (mamp418)	sHF-LIPA	CMV promoter, N-terminal His_6-FLAG	Jin et al., 2018

pcDNA3.1(-) (mamp415)	CTSD-3FLAG	CMV promoter, C-terminal 3FLAG	This study
pcDNA3.1(-) (mamp416)	CTSZ-3FLAG	CMV promoter, C-terminal 3FLAG	This study
pcDNA4.0 (mamp410)	MBTPS1-FLAG	CMV promoter, C-terminal FLAG	Chen et al., 2021
pcDNA4.0 (mamp411)	MBTPS1 (S414A)-FLAG	CMV promoter, C-terminal FLAG	Chen et al., 2021
pHAGE2-BSD (Mamp300)	TMEM251 long isoform	EF1α promoter, Blasticidin selection	This study
pHPAG2-BSD (Mamp301)	TMEM251 short isoform	EF1α promoter, Blasticidin selection	This study
pLenti6.3-BSD (Mamp317)	TMEM251 (short)-3HA	CMV promoter, Blasticidin selection	This study
pHAGE2-IRES-mCherry (Mamp149)	EGFP-RNF152	EF1α promoter, mCherry selection	Zhang et al., 2021
pLJC5 (Mamp074)	TMEM192-2XFLAG	UbC promoter	Abu-Remaileh et al., 2017<br>Addgene 102929
pLJC5 (Mamp 075)	TMEM192-3XHA	UbC promoter	Abu-Remaileh et al., 2017<br>Addgene 102930
pSpCas9(BB)-2A-Puro (PX459) (Mamp010)		CRISPR-Cas9 knockout	Ran et al., 2013<br>Addgene, 48139
Lenti-multi-CRISPR (Mamp194)		CRISPR-Cas9 knockout	Cao et al., 2016<br>Addgene 85402
psPAX2 (Mamp094)		Lentiviral packaging plasmid	Addgene 12260
pMD2.G (Mamp093)		VSV-G envelope	Addgene 12259
AG949 (Mamp372)	scFv M6P	N-terminal IL-2 signal sequence and C-terminal Fc region of Rabbit IgG	University of Geneva, This Study

**Supplementary Table 4: primers used for F0 knockout in zebrafish**	
Names	***Sequences***
tmem251 sgRNA-1	GAAATTAATACGACTCACTATATGAATTTCCGTCAGCGGATGGTTTTAGAGCTAGAAAT

tmem251 sgRNA-2	GAAATTAATACGACTCACTATAGGAGCGCAGGCAAAATGGCCGTTTTAGAGCTAGAAAT
tmem251 sgRNA-3	GAAATTAATACGACTCACTATAGAAACACCTGCAGATAGGGAGTTTTAGAGCTAGAAAT
tmem251 sgRNA-4	GAAATTAATACGACTCACTATATGATTGACACCTGAAGATGTGTTTTAGAGCTAGAAAT
gnptab sgRNA-1	GAAATTAATACGACTCACTATAGGGCTTTACCTGTGTTTCGGGTTTTAGAGCTAGAAAT
gnptab sgRNA-2	GAAATTAATACGACTCACTATAGAAGGGACTCACCGCCCTTTGTTTTAGAGCTAGAAAT
gnptab sgRNA-3	GAAATTAATACGACTCACTATAGGGCTGTGCTAACTCTTGGTGTTTTAGAGCTAGAAAT
gnptab sgRNA-4	GAAATTAATACGACTCACTATAGGAGAGGGATTCAGACTCCGGTTTTAGAGCTAGAAAT

Supplementary References:

Abu-Remaileh M, Wyant GA, Kim C, et al. Lysosomal metabolomics reveals V-ATPase- and mTOR-dependent regulation of amino acid efflux from lysosomes. *Science*. 2017;358(6364):807-813. doi:10.1126/science.aan6298

Cao J, Wu L, Zhang SM, et al. An easy and efficient inducible CRISPR/Cas9 platform with improved specificity for multiple gene targeting. *Nucleic Acids Res*. 2016;44(19):e149. doi:10.1093/nar/gkw660

Chen X, Zhang J, Liu P, et al. Proteolytic processing of secretory pathway kinase Fam20C by site-1 protease promotes biomineralization. *Proc Natl Acad Sci U S A*. 2021;118(32):e2100133118. doi:10.1073/pnas.2100133118

Jin ZC, Kitajima T, Dong W, et al. Genetic disruption of multiple α1,2-mannosidases generates mammalian cells producing recombinant proteins with high-mannose-type *N*-glycans. *J Biol Chem*. 2018;293(15):5572-5584. doi:10.1074/jbc.M117.813030

Zhang W, Yang X, Chen L, et al. A conserved ubiquitin- and ESCRT-dependent pathway internalizes human lysosomal membrane proteins for degradation. *PLoS Biol*. 2021;19(7):e3001361. Published 2021 Jul 23. doi:10.1371/journal.pbio.3001361
