# Supplementary material for: GCAF(TMEM251) regulates lysosome biogenesis by activating the mannose-6-phosphate pathway
Source: Nat Commun. 2022 Sep 12;13:5351. doi: 10.1038/s41467-022-33025-1 (PMC9468337; doi:10.1038/s41467-022-33025-1)
Supplement: Supplementary file 3 — Description of Additional Supplementary Files [file 41467_2022_33025_MOESM3_ESM.pdf]

Description of Additional Supplementary Files

File Name: Supplementary Data 1
Description: **Hits from the CRISPR screening.**

File Name: Supplementary Data 2
Description: **Raw data of RNA-seq analysis in WT and 251KO cells.**

File Name: Supplementary Data 3
Description: **Raw data of secretome analysis in WT and 251KO cells.**
