# Supplementary material for: GCAF(TMEM251) regulates lysosome biogenesis by activating the mannose-6-phosphate pathway
Source: Nat Commun. 2022 Sep 12;13:5351. doi: 10.1038/s41467-022-33025-1 (PMC9468337; doi:10.1038/s41467-022-33025-1)
Supplement: Supplementary file 7 — Reporting Summary [file 41467_2022_33025_MOESM7_ESM.pdf]

Corresponding author(s): Ming Li
Last updated by author(s): 2022/08/23

Reporting Summary

Nature Portfolio wishes to improve the reproducibility of the work that we publish. This form provides structure for consistency and transparency in reporting. For further information on Nature Portfolio policies, see our Editorial Policies and the Editorial Policy Checklist.

Statistics

For all statistical analyses, confirm that the following items are present in the figure legend, table legend, main text, or Methods section.

n/a	Confirmed	
☐	☒	The exact sample size (*n*) for each experimental group/condition, given as a discrete number and unit of measurement
☐	☒	A statement on whether measurements were taken from distinct samples or whether the same sample was measured repeatedly
☐	☒	The statistical test(s) used AND whether they are one- or two-sided<br>*Only common tests should be described solely by name; describe more complex techniques in the Methods section.*
☒	☐	A description of all covariates tested
☒	☐	A description of any assumptions or corrections, such as tests of normality and adjustment for multiple comparisons
☐	☒	A full description of the statistical parameters including central tendency (e.g. means) or other basic estimates (e.g. regression coefficient) AND variation (e.g. standard deviation) or associated estimates of uncertainty (e.g. confidence intervals)
☐	☒	For null hypothesis testing, the test statistic (e.g. *F*, *t*, *r*) with confidence intervals, effect sizes, degrees of freedom and *P* value noted<br>*Give P values as exact values whenever suitable.*
☒	☐	For Bayesian analysis, information on the choice of priors and Markov chain Monte Carlo settings
☒	☐	For hierarchical and complex designs, identification of the appropriate level for tests and full reporting of outcomes
☒	☐	Estimates of effect sizes (e.g. Cohen's *d*, Pearson's *r*), indicating how they were calculated

Our web collection on statistics for biologists contains articles on many of the points above.

Software and code

Policy information about availability of computer code

Data collection	The Western blotting images were scanned by the Licor Odyssey CLx system.Microscopy was performed with a DeltaVision system (GE Healthcare Life Sciences). The FACS was done using FACSAria III cell sorter (BD Biosciences).
Data analysis	The band intensities of Western were quantified from the raw data files using the Image Studio Ver5.2 software. Images were further cropped or adjusted using ImageJ 1.51 J8(National Institutes of Health). The Flow data was further processed with FlowJo_10.8.0. The statistics was done using Microsoft excel (Microsoft, 2016) and Prism (Graphpad, v8.0.2).

For manuscripts utilizing custom algorithms or software that are central to the research but not yet described in published literature, software must be made available to editors and reviewers. We strongly encourage code deposition in a community repository (e.g. GitHub). See the Nature Portfolio guidelines for submitting code & software for further information.

Data

Policy information about availability of data

All manuscripts must include a data availability statement. This statement should provide the following information, where applicable:

- Accession codes, unique identifiers, or web links for publicly available datasets
- A description of any restrictions on data availability
- For clinical datasets or third party data, please ensure that the statement adheres to our policy

Data supporting this study are provided within the paper and supplementary files. . The RNA-seq data generated from this study has been uploaded to NCBI with

accession code GSE209652, and the processed raw data is attached as Supplemental Table 7. The secretome data generated from this data is attached as Supplemental Table 6. Source data are provided with this paper.

Human research participants

Policy information about studies involving human research participants and Sex and Gender in Research.

Reporting on sex and gender	N/A
Population characteristics	N/A
Recruitment	N/A
Ethics oversight	N/A

Note that full information on the approval of the study protocol must also be provided in the manuscript.

Field-specific reporting

Please select the one below that is the best fit for your research. If you are not sure, read the appropriate sections before making your selection.

☒ Life sciences ☐ Behavioural & social sciences ☐ Ecological, evolutionary & environmental sciences

For a reference copy of the document with all sections, see nature.com/documents/nr-reporting-summary-flat.pdf

Life sciences study design

All studies must disclose on these points even when the disclosure is negative.

Sample size	No sample size calculation was used to predetermine the sample size. The number of independent biological replicates for cell based experiments (stated in the Figure legends) were based on experience from similar experiments in our previously published studies and consistent with the current practices in the field. A detailed description of the samples sizes is provided in the manuscript text and figure legend
Data exclusions	No data was excluded from analysis.
Replication	A detailed description of replicates is provided in the text or figure legend. Normally, at least three independent replicates were peformed.
Randomization	For cell based studies, randomization was irrelevant as cells for each experiment were processed and analyzed in parallel.
Blinding	For cell based immunoblotting experiments, operators were not blinded to the experimental groups during collection and analysis as the order of samples was required for data generation.

Reporting for specific materials, systems and methods

We require information from authors about some types of materials, experimental systems and methods used in many studies. Here, indicate whether each material, system or method listed is relevant to your study. If you are not sure if a list item applies to your research, read the appropriate section before selecting a response.

Materials & experimental systems

n/a	Involved in the study	
☐	☒	Antibodies
☐	☒	Eukaryotic cell lines
☒	☐	Palaeontology and archaeology
☐	☒	Animals and other organisms
☒	☐	Clinical data
☒	☐	Dual use research of concern

Methods

n/a	Involved in the study	
☒	☐	ChIP-seq
☐	☒	Flow cytometry
☒	☐	MRI-based neuroimaging

Antibodies

Antibodies used	The following primary antibodies were used for western blotting in this study: rabbit anti-GFP (1:3000, TP401, Torrey Pines Biolabs), mouse anti-actin (1:5000, Proteintech), mouse anti-GAPDH (1:2000, Proteintech), rabbit anti-CTSD (1:1000, Proteintech), rabbit anti-

	Golgin160 (1:1000, Proteintech), rabbit anti-p62 (1:2000, Proteintech), rabbit anti-LC3 (1:2000, Proteintech), rabbit anti-IGF2R (CI-MPR) (1:2000, Proteintech), mouse anti-HA (1:500, 16B12, BioLegend), mouse anti-CTSC (1:500, D-6, Santa Cruz Biotechnology), mouse anti- SREBF2/SREBP2 (1:500, 1C6, Santa Cruz Biotechnology), rabbit anti-FLAG (1:2000, Millipore-Sigma), rabbit anti-LAPTM4A (1:1000, HPA, Millipore-Sigma), rabbit anti-TMEM251 (1:1000, Millipore-Sigma), mouse anti-V5 (1:3000, Invitrogen,460705), rabbit anti-ATF6 (1:1000, Proteintech), rabbit anti-EGFR (1:2000, a generous gift from Dr. Stuart Decker at the University of Michigan). The following secondary antibodies were used in this study: goat anti-mouse IRDye 680LT(LI-COR Biosciences,926-68020, 1:10000), goat anti-mouse IRDye 800CW(LI-COR Biosciences,926-32210, 1:10000) , goat anti-rabbit IRDye 680LT(LI-COR Biosciences,926-68021,1:10000), goat anti-rabbit IRDye 800CW(LI-COR Biosciences,926-32211,1:10000). Streptavidin secondary antibodies (IRDye® 800CW Streptavidin, LI-COR Biosciences,926322230, 1:2000).
Validation	Antibodies used in this study are commercially available and validated by the companies. Validation information for each antibody can be found on the manufacturer's website.

Eukaryotic cell lines

Policy information about cell lines and Sex and Gender in Research

Cell line source(s)	Cell lines used in this study are listed in Table S3. HEK293 (CRL-1573), HEK293T (CRL-3216), and HeLa (CCL-2) were purchased from ATCC.
Authentication	Cell lines used in this study were not authenticated by us.
Mycoplasma contamination	All cells were tested negative for mycoplasma.
Commonly misidentified lines (See ICLAC register)	No commonly misidentified cell lines were used in this study.

Animals and other research organisms

Policy information about studies involving animals; ARRIVE guidelines recommended for reporting animal research, and Sex and Gender in Research

Laboratory animals	Zebrafish were raised to 7 days following standard zebrafish husbandry guidelines.
Wild animals	No wild animals were used in the study.
Reporting on sex	Sex was not considered in the experiments designed.
Field-collected samples	No field collected samples were used in the study.
Ethics oversight	All experiments were conducted in accordance with the guidelines approved by the Institutional Committee on the Use and Care of Animals, University of Michigan.

Note that full information on the approval of the study protocol must also be provided in the manuscript.

Flow Cytometry

Plots

Confirm that:

☒ The axis labels state the marker and fluorochrome used (e.g. CD4-FITC).

☒ The axis scales are clearly visible. Include numbers along axes only for bottom left plot of group (a 'group' is an analysis of identical markers).

☒ All plots are contour plots with outliers or pseudocolor plots.

☒ A numerical value for number of cells or percentage (with statistics) is provided.

Methodology

Sample preparation	Cells were washed with 1XPBS and trypsinized until all cells were dissociated from the dishes. Dissociated cells were neutralized with DMEM containing 10% serum media and pelleted at 300 g for 3 minutes. Cells were resuspended in ice-cold 1XPBS.
Instrument	FACSAria III cell sorter (BD Biosciences).
Software	Flowjo
Cell population abundance	For Crisper screening, About 1.5 x 100000000 Transduced cells were subjected to FACS, and 1-1.5% cells with a high GFP/ mCherry ratio were collected.

Gating strategy | The top 1-1.5% cells with a high GFP/mCherry ratio were collected.

☒ Tick this box to confirm that a figure exemplifying the gating strategy is provided in the Supplementary Information.
